# Trust Me, I’m a Chatbot: How Artificial Intelligence in Health Care Fails the Turing Test

**DOI:** 10.2196/16222

**Published:** 2019-10-28

**Authors:** John Powell

**Affiliations:** 1 Nuffield Department of Primary Care Health Sciences Medical Sciences Division University of Oxford Oxford United Kingdom

**Keywords:** artificial intelligence, machine learning, medical informatics, digital health, ehealth, chatbots, conversational agents

## Abstract

Over the next decade, one issue which will dominate sociotechnical studies in health informatics is the extent to which the promise of artificial intelligence in health care will be realized, along with the social and ethical issues which accompany it. A useful thought experiment is the application of the Turing test to user-facing artificial intelligence systems in health care (such as chatbots or conversational agents). In this paper I argue that many medical decisions require value judgements and the doctor-patient relationship requires empathy and understanding to arrive at a shared decision, often handling large areas of uncertainty and balancing competing risks. Arguably, medicine requires wisdom more than intelligence, artificial or otherwise. Artificial intelligence therefore needs to supplement rather than replace medical professionals, and identifying the complementary positioning of artificial intelligence in medical consultation is a key challenge for the future. In health care, artificial intelligence needs to pass the implementation game, not the imitation game.

Over the last two decades, the concerns of digital health researchers interested in the social impact of the internet have evolved as the technology has matured and new tools have emerged. From a sociotechnical perspective, there were initial preoccupations with the impact of a new, uncontrolled form of mass communication, alongside concerns with the quality of unregulated online information and threats to professions, with medical professionals in particular fearing a loss of authority [1-3]. As Web2.0 developments took hold and the public became producers as well as consumers of health information, researchers began to identify the benefits of online peer-to-peer communication and the sharing of information in virtual communities, social media, and increasingly on health ratings sites [4-7]. With the mass uptake in smartphones, the subsequent rapid developments in mobile health, and the explosion in health apps, we are now exploring the value of low-cost, patient-centered interventions delivered directly to consumers [8,9]. In addition, we are also gaining a better understanding of the limitations and key issues in their implementation, such as nonadoption and abandonment [10]. As the number one journal in this field, the Journal of Medical Internet Research continues to reflect and illuminate all these debates.

For those of us studying the social science of digital technology in health and health care, one area of research is likely to dominate the next decade: the extent to which the promise of artificial intelligence (AI) in health care will be realized, and the social and ethical issues which accompany it [11-13]. Broadly speaking, we can identify two current strands in the use of AI in health care. Firstly, there are data-facing applications which use techniques such as machine learning and artificial neural networks to derive new knowledge from large datasets, such as improving diagnostic accuracy from scans and other images [14]. Secondly, there are user-facing applications and intelligent agents which interact with people in real-time, using inferences to provide advice or instruction based on probabilities which the tool can derive and improve over time, such as a chatbot substituting or complementing a health care consultation with a patient [15]. In this article I focus on the latter to consider the approaches of these chatbots, or “robot doctors,” to medical consultation, and specifically the extent to which these technologies will ever pass the celebrated Turing test.

Alan Turing, the British mathematician and theoretical computer scientist, is widely regarded as the founding father of AI. He proposed that for a machine to be considered intelligent it should provide responses to a blinded interrogation that are indistinguishable from those given by a human comparator [16]. In other words, the interrogator should not be able to tell whether the machine or the human was responding. If we extrapolate this thought experiment to current health care, we can pose the question of whether AI-based medical consultations (conversational agents and medical chatbots) will ever be considered intelligent by Turing’s standard. Of course, context is important, and if a patient is asking a simple factual question that requires a binary response, for example, then even current AI systems can mimic a human interlocutor with high accuracy. However, we know that medical consultations are complex [17], that many medical decisions require value judgements, and that the doctor-patient relationship requires empathy and understanding to arrive at a shared decision [18]. The practice of medicine is as much an art as a science, and patients may choose a path which is not necessarily the one that logic would determine. Even the pioneers of evidence-based medicine defined their normative approach as:

the conscientious and judicious use of current best evidence from clinical care research in the management of individual patients [19].

Conscience and the ability to weigh competing personal values are not strengths of AI. A key skill for medical professionals is the ability to deal with uncertainty alongside considering patients’ preferences. What doctors often need is wisdom rather than intelligence, and we are a long way away from a science of artificial wisdom.

It is doubtful whether AI will ever pass the Turing test for complex medical consultations, but this is to misunderstand the place of AI in future medical care. AI should complement rather than replace medical professionals. As various studies into the future of work have shown, automation in the workplace will not eliminate all human tasks [20]. Chatbot approaches have many potential benefits, including the potential to allow clinicians to have more time for delivering empathic and personalized care [15]. Perhaps, as a senior clinical informatics leader in the UK has suggested, “AI will allow doctors to be more human” [13]. However, as has been well established for many innovations in health care, especially digital ones, the key challenges for health systems seeking to harness the benefits of the technology are not just related to its effectiveness but also to the wider issues of its integration and implementation [10,12,21]. We need to understand how to integrate the tools and practices of AI within the work and culture of professionals and organizations, to investigate factors related to adoption, nonadoption, and abandonment [10,12], and investigate the work required to sustain innovation [22]. Factors which will influence the implementation of AI tools include those related to people, such as professional and public attitudes, trust, existing work practices, training needs, and the risks of deskilling and disempowerment; those related to the health system, such as leadership and management, the positioning of clinical responsibility and accountability, and the possibility of harm, alongside issues of regulation and service provision (including scalability and the possibility of providing two-tier services with or without AI); those related to the data, such as issues of data security, privacy, consent and ownership; and those related to the tool itself, such as transparency of the algorithm, issues of reliability and validity, and algorithmic bias [12,21,23]. To take an example, in an early study of an algorithm-based triage tool in primary care, we showed that physicians lacked trust in the ability of the machine to take clinical risks and worried about issues of governance and accountability, such that the sensitivity of the tool, in terms of the urgency of triage, was consistently set at a threshold which would increase urgent clinical workload rather than reduce it [24].

Identifying the complementary positioning of AI tools in health care in general, and in particular for their use in the medical consultation, is a key challenge for the future. We need to understand how to integrate the precision and power of AI tools and practices with the wisdom and empathy of the doctor-patient relationship. In health care, it is more important that artificial intelligence passes the implementation game rather than the imitation game.

## Abbreviations

AI: artificial intelligence

## References

[1] Hardey M (2001). Doctor in the house: the Internet as a source of lay health knowledge and the challenge to expertise. Sociology of Health & Illness.

[2] Eysenbach G, Powell J, Kuss O, Sa E (2002). Empirical studies assessing the quality of health information for consumers on the world wide web: a systematic review. JAMA.

[3] Ziebland S (2004). The importance of being expert: the quest for cancer information on the Internet. Soc Sci Med.

[4] Hardey M (2001). 'E-health': the internet and the transformation of patients into consumers and producers of health knowledge. Info., Comm. & Soc.

[5] Powell J, McCarthy N, Eysenbach G (2003). Cross-sectional survey of users of Internet depression communities. BMC Psychiatry.

[6] Eysenbach G, Powell J, Englesakis M, Rizo C, Stern A (2004). Health related virtual communities and electronic support groups: systematic review of the effects of online peer to peer interactions. BMJ.

[7] van Velthoven MH, Atherton H, Powell J (2018). A cross sectional survey of the UK public to understand use of online ratings and reviews of health services. Patient Educ Couns.

[8] Powell J, Hamborg T, Stallard N, Burls A, McSorley J, Bennett K, Griffiths KM, Christensen H (2012). Effectiveness of a web-based cognitive-behavioral tool to improve mental well-being in the general population: randomized controlled trial. J Med Internet Res.

[9] Rathbone AL, Clarry L, Prescott J (2017). Assessing the Efficacy of Mobile Health Apps Using the Basic Principles of Cognitive Behavioral Therapy: Systematic Review. J Med Internet Res.

[10] Greenhalgh T, Wherton J, Papoutsi C, Lynch J, Hughes G, A'Court C, Hinder S, Fahy N, Procter R, Shaw S (2017). Beyond Adoption: A New Framework for Theorizing and Evaluating Nonadoption, Abandonment, and Challenges to the Scale-Up, Spread, and Sustainability of Health and Care Technologies. J Med Internet Res.

[11] Vayena E, Blasimme A, Cohen IG (2018). Machine learning in medicine: Addressing ethical challenges. PLoS Med.

[12] Shaw J, Rudzicz F, Jamieson T, Goldfarb A (2019). Artificial Intelligence and the Implementation Challenge. J Med Internet Res.

[13] (2019). Academy of Medical Royal Colleges.

[14] Shen J, Zhang CJP, Jiang B, Chen J, Song J, Liu Z, He Z, Wong SY, Fang P, Ming W (2019). Artificial Intelligence Versus Clinicians in Disease Diagnosis: Systematic Review. JMIR Med Inform.

[15] Palanica A, Flaschner P, Thommandram A, Li M, Fossat Y (2019). Physicians' Perceptions of Chatbots in Health Care: Cross-Sectional Web-Based Survey. J Med Internet Res.

[16] Turing AM (1950). Computing Machinery and Intelligence. Mind, New Series.

[17] Innes AD, Campion PD, Griffiths FE (2005). Complex consultations and the 'edge of chaos'. Br J Gen Pract.

[18] Barry MJ, Edgman-Levitan S (2012). Shared decision making - The pinnacle of patient-centered care. N Engl J Med.

[19] Sackett DL, Rosenberg WMC, Gray JAM, Haynes RB, Richardson WS (1996). Evidence based medicine: what it is and what it isn't. BMJ.

[20] Autor DH (2015). Why Are There Still So Many Jobs? The History and Future of Workplace Automation. Journal of Economic Perspectives.

[21] Cresswell KM, Bates DW, Sheikh A (2013). Ten key considerations for the successful implementation and adoption of large-scale health information technology. J Am Med Inform Assoc.

[22] Pope C, Halford S, Turnbull J, Prichard J, Calestani M, May C (2013). Using computer decision support systems in NHS emergency and urgent care: ethnographic study using normalisation process theory. BMC Health Serv Res.

[23] Greenhalgh T, Robert G, Macfarlane F, Bate P, Kyriakidou O (2004). Diffusion of innovations in service organizations: systematic review and recommendations. Milbank Q.

[24] Poote AE, French DP, Dale J, Powell J (2014). A study of automated self-assessment in a primary care student health centre setting. J Telemed Telecare.

